# IL-10 partly mediates the ability of MSC-derived extracellular vesicles to attenuate myocardial damage in experimental metabolic renovascular hypertension

**DOI:** 10.3389/fimmu.2022.940093

**Published:** 2022-09-20

**Authors:** Yamei Jiang, Siting Hong, Xiangyang Zhu, Lei Zhang, Hui Tang, Kyra L. Jordan, Ishran M. Saadiq, Weijun Huang, Amir Lerman, Alfonso Eirin, Lilach O. Lerman

**Affiliations:** ^1^ Division of Nephrology and Hypertension, Mayo Clinic, Rochester, MN, United States; ^2^ Department of Cardiovascular Diseases, Mayo Clinic, Rochester, MN, United States

**Keywords:** renovascular hypertension, extracellular vesicles, interleukin-10, metabolic syndrome, myocardial damage, mesenchymal stem cell

## Abstract

Extracellular vesicles (EVs) obtain properties of immunomodulation and tissue repair from their parental mesenchymal stem cells (MSCs), and upon delivery may be associated with fewer adverse events. EVs derived from adipose-tissue MSCs restored kidney function by attenuating kidney inflammation in a swine model of metabolic syndrome (MetS) and renal artery stenosis *via* anti-inflammatory pathways. EVs also ameliorated myocardial injury in renovascular hypertension (RVH) secondary to inflammation in cardiorenal disease, but the mechanisms regulating this effect are unknown. We hypothesize that the anti-inflammatory cytokine interleukin (IL)-10 mediates the reparative effects of EVs on cardiovascular complications in a preclinical swine model with coexisting MetS and RVH. Twenty-three pigs established as Lean controls or RVH models were observed for 16 weeks. At 12 weeks RVH subgroups received an intrarenal delivery of 10^11^ either wildtype (WT) EVs or EVs after IL-10 knockdown (KD) (RVH+WT-EVs or RVH+IL-10-KD-EVs, respectively). Cardiac and renal function were studied *in-vivo* and myocardial tissue injury *in-vitro* 4 weeks later. RVH pigs showed myocardial inflammation, fibrosis, and left ventricular diastolic dysfunction. WT-EVs attenuated these impairments, increased capillary density, and decreased myocardial inflammation *in-vivo*. *In-vitro*, co-incubation with IL-10-containing WT-EVs decreased activated T-cells proliferation and endothelial cells inflammation and promoted their migration. Contrarily, these cardioprotective effects were largely blunted using IL-10-KD-EVs. Thus, the anti-inflammatory and pro-angiogenic effects of EVs in RVH may be partly attributed to their cargo of anti-inflammatory IL-10. Early intervention of IL-10-containing EVs may be helpful to prevent cardiovascular complications of MetS concurrent with RVH.

## Introduction

Renal artery stenosis (RAS), mainly caused by atherosclerosis (1), is a common etiology of renovascular hypertension (RVH) (2). RAS not only results in hemodynamic disorders and deteriorating renal function, but is also exacerbated by systemic diseases including metabolic syndrome (MetS) and diabetes mellitus (3). Conversely, all of these risk factors are crucial contributors to atherosclerosis (4) and cardiovascular disease (CVD) (5), a leading cause of death in the U.S (6, 7). Therefore, strategies to blunt kidney and cardiac damage in subjects with RVH with metabolic perturbation are in dire need.

Mesenchymal stem cells (MSCs) are self-renewable, multipotent cells with therapeutic and repair functions, which have shown promise in clinical trials for cardiac and renal repair (8–10). Most of the MSC-mediated beneficial effects are attributed to their paracrine activity (11). Among their paracrine factors, MSC-derived extracellular vesicles (EVs) carry cargo that resembles the composition of their parental cells and show similar therapeutic functions while circumventing the risk for rejection and tumorigenesis (12). Our lab has found that intrarenal delivery of adipose MSC-derived EVs in a porcine model of RVH improved renal function and alleviated myocardial remodeling (13). In addition, we found that IL-10 knock-down (KD) in EVs blunted their renoprotective effects, suggesting that the salutary effects of EVs on the kidney may be attributed to their content of anti-inflammatory cytokines (14). However, whether IL-10 in the adipose MSC-derived EVs modulate their effects on the heart in pigs with RVH coexisting with MetS remains unknown.

Therefore, the present study took advantage of a preclinical swine model of RVH in the milieu of MetS, mimicking the clinical situation, to test the hypothesis that IL-10 regulates the reparative effect of MSC-derived EVs on cardiac function.

## Materials and methods

### Animal experiments

The study was approved by the Mayo Clinic Institutional Animal Care and Use Committee. Twenty-three 3-months old female pigs were studied for 16 weeks. At baseline, 17 pigs were fed with a high-cholesterol/high-carbohydrate diet (15), and on week 6, underwent RAS surgery to induce RVH as described (16). Briefly, 0.25g tiletamine hydrochloride/zolazepam hydrochloride (Telazol^®^, Zoetis, Kalamazoo, MI) and 0.5g xylazine (Xylamed, VetOne, Bimeda-MTC Animal Health, Cambridge, ON, Canada) were used for intramuscular anesthesia, followed by intravenous ketamine (0.2 mg/kg/min, Ketaset, Zoetis, Kalamazoo, MI) and xylazine (0.03 mg/kg/min) for maintenance. Then unilateral RAS was induced by placing a local irritant coil in the main right renal artery under fluoroscopic guidance (Siemens, Munich, Germany) (17). Then, abdominal adipose tissue (~2g) was collected from each pig *via* biopsy to harvest autologous MSCs and isolate their EVs. MSCs from five RVH pigs had been pretreated with siRNA prior to EV collection to knock down IL-10. Six other pigs (Lean) were fed a standard pig chow throughout the 16 weeks and underwent a sham operation to serve as controls.

On week 12, angiography was used to assess the degree of stenosis in all pigs, and mean arterial pressure (MAP) was measured using a catheter through a cannulated carotid artery. Then the 17 RVH pigs were divided into three groups: RVH (n=6), RVH+wildtype (WT)-EVs (n=6), and RVH+IL-10-KD EVs (n=5). Both types of EVs were suspended (1×10^11^) in phosphate-buffered saline (PBS) and injected into the stenotic kidney over 5 min through a 5-F catheter positioned proximal to the stenosis. Lean and RVH pigs received PBS vehicle.

On week 16, all pigs were anesthetized to repeat renal angiography and MAP measurement, and study cardiac and renal function. Systemic blood samples were collected for serum creatinine (SCr) and plasma renin activity (PRA) levels (MAK157, Millipore-Sigma, Burlington, MA). A few days later, the animals were euthanized with IV sodium pentobarbital (100mg/kg). The hearts were dissected, and sections were frozen in liquid nitrogen or preserved in formalin for further studies. Coronary arteries were also collected for *ex vivo* studies.

### Cardiac and renal function

On week 16, cardiac function including left ventricular (LV) ejection fraction (EF), cardiac output (CO), systemic vascular resistance (SVR), E/A ratio (an index of diastolic function), bilateral single-kidney glomerular filtration rate (GFR), and single-kidney volume were assessed using multi-detector computed tomography (MDCT, Somatom Sensation-128, Siemens), and analyzed with MATLAB 7.10. as previously described (13).

### EVs characterization and delivery

MSCs were isolated from abdominal subcutaneous adipose tissue using collagenase-H (11074032001, Millipore-Sigma) and cultured in advanced Eagle minimal essential medium (MEM, 12492013, Gibco, Waltham, MA) supplemented with 5% platelet lysate (Mill Creek Life Sciences, Rochester, MN) at 37°C with 5% CO_2_, as previously reported (18, 19). Common MSC markers were identified through immunofluorescent (IF) staining and flow cytometry. Trans-differentiation into adipocytes, chondrocytes, and osteocytes was also confirmed as previously described (14).

We used siRNA (Ambion, Life Technologies) to silence the expression of IL-10 in MSCs of the RVH+IL-10-KD EVs group. Briefly, when MSCs were about 70% confluent, we changed the medium to Opti-MEM medium (31985070, Gibco) supplemented with siRNA lipofectamine (13778075, ThermoFisher, Waltham, MA) guided by instructions. Twenty-four hrs later, we changed the medium to medium 199 (m199, 11150059, Gibco) without fetal bovine serum (FBS) to increase EV release, and 48hrs later, the supernatant was collected to harvest EVs through size-exclusion chromatography (20). Firstly, the supernatant was centrifuged at 2,000×g for 20min to remove cells and debris and then was concentrated into a small volume using centrifugal filter devices (Centricon Plus-70, MilliporeSigma), followed by EV isolation with an appropriate qEV column (iZON Science). Ultimately, another centrifugal filter concentrated the EVs further. For the RVH+WT-EVs group, the medium of MSCs was changed to m199 at 70% confluency and WT-EVs were harvested from supernatant 48hrs later. To characterize EVs, nanoparticle tracking analysis (NTA, NanoSight NS300, Malvern Panalytical Ltd, Malvern, UK) was used to assess their concentration and size distribution. IL-10 mRNA expression was determined in MSCs and EVs. After labeling with red fluorescence PKH26, EVs were delivered into the stenotic renal artery through a catheter under fluoroscopic guidance. The pigs were then allowed to recover and returned to their housing.

### Heart tissue experiments

EV tracking was performed in 10μm frozen heart tissue sections by counting red EV aggregates in 10 random fields of each section under fluorescent microscopy. Hematoxylin-eosin (H&E) and Trichrome Masson’s staining (#9179, Newcomer supply, Middleton, WI, USA) were performed to investigate the capillary density and fibrosis. Briefly, heart tissue was fixed in 4% paraformaldehyde and embedded in paraffin, followed by sectioning at 4μm thickness. In each slide, 10 fields were selected randomly and results from all fields were averaged. Cellular infiltration with macrophages and regulatory T-cells (Treg) was evaluated by IF staining with primary antibodies against CD68 (MA5-13324), inducible nitric oxide synthase (iNOS, PA1-036), Arginase-1 (Arg-1, sc-20150, Santa-Cruz), CD4 (NBP1-19371, Novus Biologicals, Centennial CO), CD25 (ab207895, Abcam) and Foxp3 (ab20034, Abcam).

Inflammatory cytokines in heart tissues and IL-10 mRNA expression in MSCs and EVs were assessed by real-time polymerase chain reaction (RT-PCR). Briefly, total RNA was extracted from tissue or cells using the kit (#AM1556, Life Technologies). Then, SuperScript VILO cDNA synthesis kit (#11754-050) was used to obtain cDNA. Relative quantitative PCR utilized Taqman assays with the following probes: IL-1β (Ss03393804, ThermoFisher), IL-6 (Ss03384604), TNF-α (Ss03391318), MCP-1 (Ss03394377), IL-10 (Ss03382372) and GAPDH (Ss03375435). Fold change of gene expression was calculated using the 2^-△△CT^ method.

Western blot analysis was performed to detect vascular endothelial growth factor (VEGF) protein levels in the tissue. Briefly, the myocardial tissue was lysed with RIPA buffer (9806, Cell Signaling Technology, Danvers, MA, USA). Protein concentration was detected using a BCA protein assay kit (23227, ThermoFisher). After being separated by 4-20% precast gel (5671095, BIO-RAD, Hercules, CA, USA), the protein samples were then transferred to PVDF membranes (PB5310, Invitrogen) and blocked in 5% BSA (37520, ThermoFisher). The membrane was incubated with primary antibodies anti-VEGF (sc-7269, Santa-Cruz) and anti-GAPDH (ab8245, Abcam) overnight at 4°C followed by incubation with a secondary antibody for 1hrs. Enhanced chemiluminescence (34096, ThermoFisher) was used to visualize the blots.

### *In vitro* T-cells proliferation

The immunomodulatory effects of EVs with or without IL-10 were evaluated in stimulated T-cells (21–23). Fresh human CD3+ Pan T-cells (4W-350, Lonza, Walkersville, MD, USA) were cultured in Roswell Park Memorial Institute (RPMI) 1640 medium (10-040-CM, Corning Life Sciences, Corning, NY, USA) supplemented with 10% FBS for 6hrs after been thawed. Next, the cells were prelabelled with a 1μM CellTrace Far Red (CTFR) following the manufacturer’s instructions (C34572, Invitrogen). Then cells were seeded in 6-well plates (1×10^6^/well), stimulated with 5μg/ml concanavalin-A (ConA, C2010, Sigma-Aldrich, St. Louis, MO), and cocultured with vehicle (PBS), WT-EVs or IL-10-KD EVs (T-cells to EVs 1:5000 ratio). In these *in-vitro* experiments, we included two additional negative control groups of cells incubated with EVs treated with an empty vector siRNA (NC-EVs, AM4611, Ambion, Life Technologies) or lipofectamine (Lipo-EVs, 13778075, ThermoFisher). Three days later, the cell pellets were collected, washed with FACS buffer (PBS with 1% BSA), and stained with a Live/Dead Violet Viability/Vitality kit (L34958, Invitrogen) at ambient temperature for 30min. Finally, the cells were washed and studied with Flow Cytometry (Amnis^®^ FlowSight^®^ Image Flow Cytometry, Luminex, Chicago, USA) to test their proliferation. Negative controls included T-cells alone, with NC-EVs, or with Lipo-EVs, and positive controls included T-cells with ConA stimulation. Six replicates were conducted for each group.

### Endothelial cell isolation, identification, and migration

Primary coronary artery endothelial cells (CAECs) were isolated from Lean and RVH pigs. Briefly, the right coronary artery was procured from the heart and a plastic tube was inserted and fixed at one end to establish an infusion channel. The inner wall of the artery was washed by PBS three times, followed by a slow infusion of 0.1% Collagenase type-I (17100015, Gibco) until it outflowed from the other end of the artery, after which the artery was ligated at the end and incubated at 37°C for 30min in a 10cm dish. The arterial ligation was opened and flushed several times with endothelial medium (CC-5036, Lonza). The cellular suspension was filtered through a 100μm cell strainer and centrifuged for 5min at 1000 RPM to get pellets and resuspended in the same culture medium, cultured in a 37°C CO_2_ incubator.

Immunocytochemistry staining was conducted for the identification of CAECs with the markers CD31 (MCA1747, BIO-RAD, Hercules, California, USA), VEGF (sc-7269, Santa-Cruz), as well as the negative marker alpha-smooth muscle actin (α-SMA, ab7817, Abcam).

CAECs were then used for migration assays. CAECs from Lean and RVH pigs are subsequently co-incubated with vehicle, NC-EVs, Lipo-EVs, WT-EVs or IL-10-KD EVs. For migration, the cells were seeded in a 24-well plate, treated with EVs (at a 1:5000 ratio), and placed in an incubator until 80% confluency in the monolayer. Then, a wound was created in the center of each well by the auto-scratch wound-making tool (BioTek, Agilent Technologies, Pittsburgh, PA, USA). After a medium change, the cells were placed in Cytation-5 (BioTek) at 37°C with 5% CO_2_ for 48hrs to study their migration, indexed by their ability to migrate across the scratch.

### Statistical analysis

All data were presented as mean ± SD. Normality distribution was detected first. For two groups comparison, student’s *t*-test or a nonparametric test was applied as applicable. For four groups comparison, one-way ANOVA was conducted followed by either the Bonferroni *post-hoc* if normally distributed or Two-stage step-up method of Benjamini, Krieger, and Yekutieli *post-hoc* in case of non-normal distribution. GraphPad Prism software (version 9.0.1, San Diego, CA) was used for statistical analysis. A *p*-value<0.05 was considered statistically significant.

## Results

### MSCs and EVs characterization

**Figure 1:**
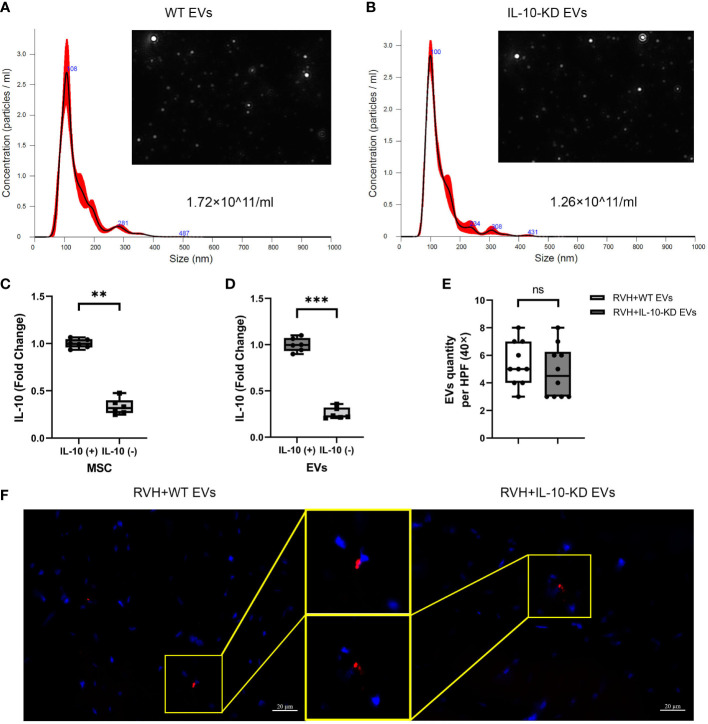
Characterization and tracking of swine MSC-derived EVs. **(A, B)**. NTA showed the size distribution and quantity of EVs from 15×10^6^ MSCs and IL-10-KD MSCs. **(C, D)**. qPCR detected the IL-10 mRNA expression in MSCs and their daughter EVs. n = 6 **(E, F)**. EVs tracking in the heart sections and comparable numbers of PKH-26-labeled EVs between RVH+EVs and RVH+IL-10-KD EVs. Blue is DAPI-stained cell nuclei. Scale bar = 20µm. Data are expressed as mean ± SD. **p < 0.01, ***p < 0.001 between groups (Student’s *t*-test following normality and log-normality tests). MSCs, mesenchymal stem cells; EVs, extracellular vesicles; NTA, Nanoparticle Tracking Analysis; RVH, renovascular hypertension; KD, knockdown. ns, no significance.

We have previously shown that isolated MSCs express typical markers CD34, CD90, and CD44 and are capable of transdifferentiating into adipocytes, osteocytes, and chondrocytes (24, 25). NTA revealed that 15×10^6^ MSCs (comparable to their *in-vivo* dose) cultured for 72hrs released about 1×10^11^ EVs/ml. The size distribution of both WT-EVs and IL-10-KD EVs showed that most of them were exosomes (~150nm) and a smaller fraction included small micro-vesicles (~200nm) (**Figures 1A, B**).

siRNA treatment significantly decreased IL-10 mRNA expression in both IL-10-KD MSCs and their daughter EVs (**Figures 1C, D**). Red fluorescent signals of WT-EVs and IL-10-KD-EVs labeled with PKH26 were detected in frozen heart sections (**Figure 1E**) and quantitative analysis (**Figure 1F**) found similar myocardial retention in both groups.

### Renal and cardiac function

**Figure 2:**
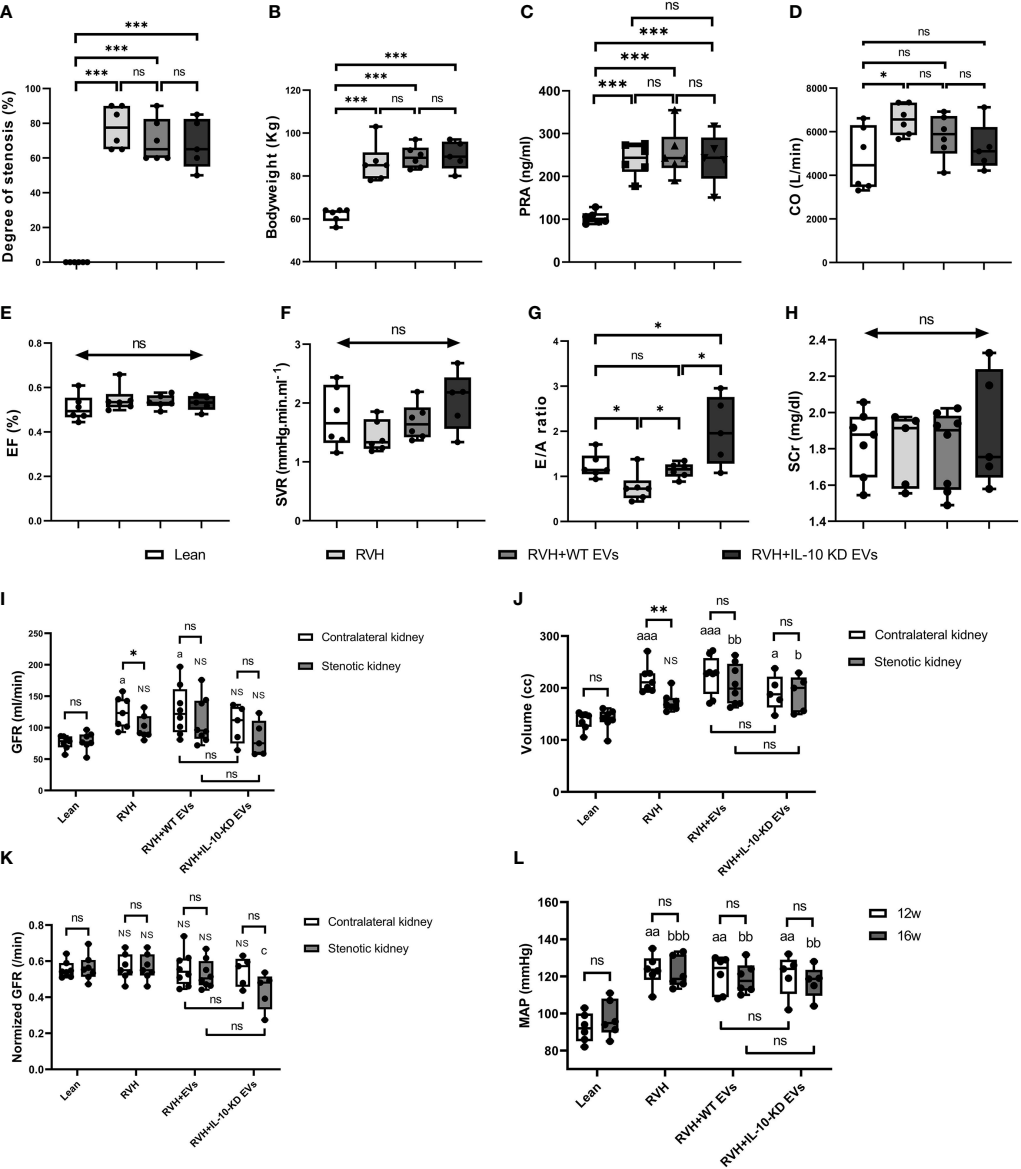
Systemic characteristics, cardiac and renal function in the study groups. Systemic characteristics, including the **(A)** degree of stenosis, **(B)** bodyweight, and **(C)** PRA at week 16. **(D–H)** Cardiac function and SCr at week 16. Data are mean ± SD. *p < 0.05, **p < 0.01, ***p < 0.001, double-headed arrow with ‘ns’ indicate no statistical significance among the included groups (One-way ANOVA followed by either the Bonferroni posttest or Two-stage step-up method of Benjamini, Krieger and Yekutieli posttest as appropriate). **(I–K)** Single-kidney GFR, kidney volume and normalized GFR in all groups. ‘ns’ no significance, *p < 0.05, **p < 0.01: compared two kidneys in particular; ‘NS’ no significance, ^a^ *p *< 0.05, ^aaa^ *p *< 0.001, ^b^ *p* < 0.05, ^bb^ *p* < 0.01 compared with single-kidney in the Lean group; ^c^ *p* < 0.05 compared to stenotic kidneys in RVH group (Student’s *t*-test when normally distributed, otherwise a nonparametric test; One-way ANOVA followed by an appropriate posttest test). **(L)** MAP was elevated at 12 and 16 weeks in all RVH groups. ^aa^ *p* < 0.01 compared with Lean group at week 12, ^bb^ *p* < 0.01, ^bbb^ *p* < 0.001 compared with Lean group at week 16 (One-way ANOVA followed by posttest). PRA, plasma renin activity; CO, Cardiac output; EF, ejection fraction; SVR, systemic vascular resistance; E/A ratio, the ratio of peak velocity flow from left ventricular relaxation in early diastole (E wave) to peak velocity flow in late diastole caused by atrial contraction (A wave), a marker of the left ventricle function; SCr, serum creatinine; GFR, glomerular filtration rate; MAP, mean arterial pressure.

**Figure 2** shows the systemic characteristics and renal and cardiac function of all pigs at week 16. All RVH pigs developed significant and similar (*p*>0.05 among RVH groups) RAS, obesity, and elevated PRA (**Figures 2A** **–C**). CO was significantly increased only in untreated RVH pigs (*p*=0.022 vs. Lean) but did not differ from other RVH groups (**Figure 2D**). EF and SVR showed no difference among groups (**Figures 2E, F**). E/A ratio was lower in RVH, normalized in RVH+EVs, but markedly increased in IL-10-KD EVs pigs compared with other groups (**Figure 2G**). There was no statistically significant difference in systemic SCr among the groups (*p*=0.870) (**Figure 2H**). GFR was significantly lower in stenotic compared with contralateral RVH kidneys (*p*=0.043), whereas in Lean (right vs. left) and EV-treated RVH pigs GFR of the two kidneys showed no significant difference (*p*>0.05). GFR was also higher in contralateral RVH (*p*=0.029) and RVH+WT-EVs (*p*=0.013) kidneys compared with Lean single-kidney GFR. WT-EVs and IL-10-KD-EVs did not differ in GFR (**Figure 2I**). The stenotic kidneys were much smaller than the contralateral ones in the RVH group (*p*=0.004, **Figure 2J**). GFR normalized per unit kidney volume (**Figure 2K**) was unchanged among the groups, except for being lower in the stenotic kidneys of RVH+IL-10-KD-EVs than in the RVH stenotic kidneys (but vs. Lean). MAP was significantly and similarly increased in all RVH groups both at 12 and 16 weeks (*p*<0.01 vs. Lean, **Figure 2L**).

### IL-10-KD-EVs showed weaker anti-inflammatory properties than WT-EVs

**Figure 3:**
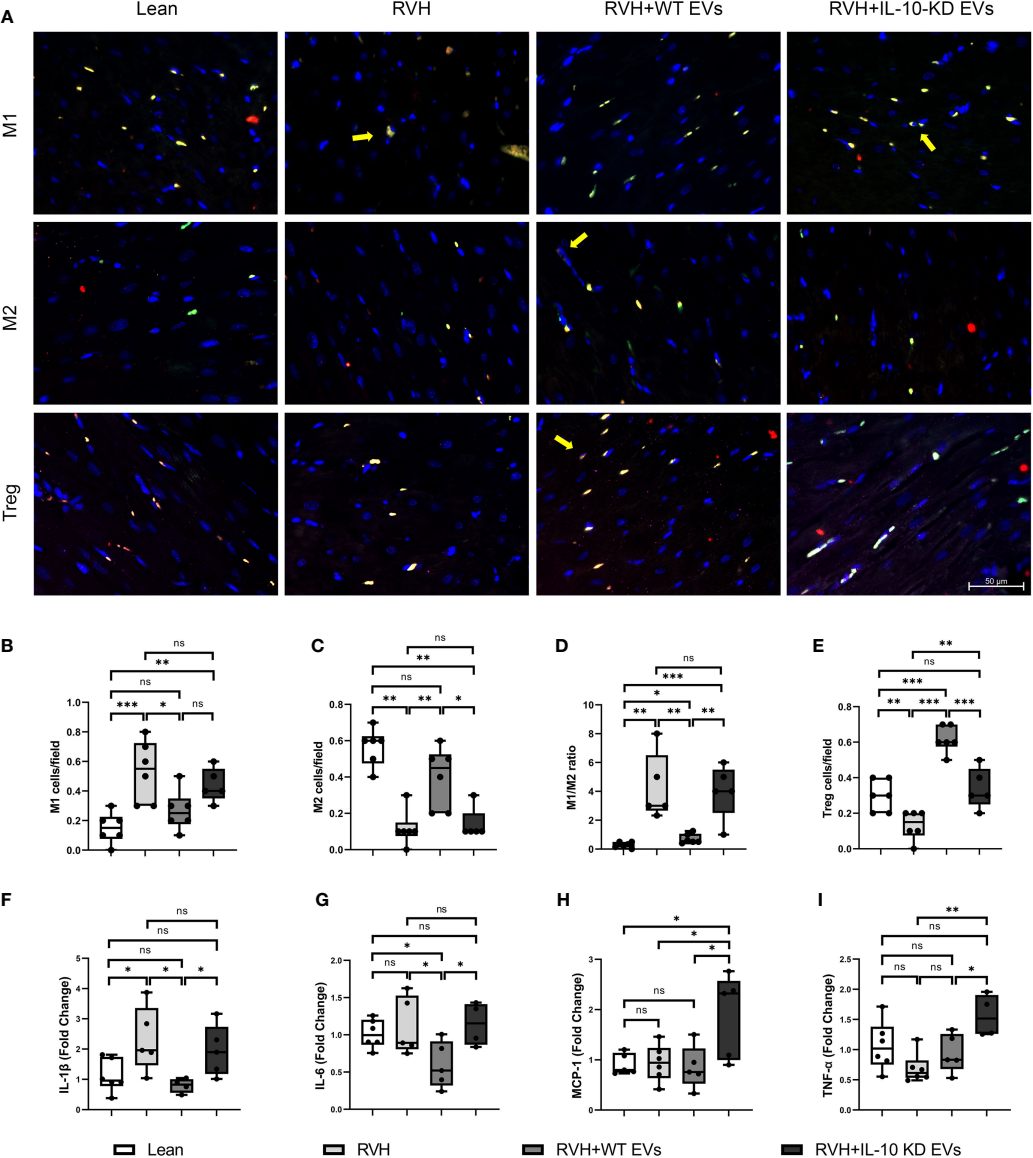
EVs modulated inflammation in the heart tissue. **(A)** Representative images (40×) of immunofluorescence staining showed that EVs induced macrophage polarization towards M2 phenotype and increased Tregs while IL-10-KD EVs had opposite effect. M1 [CD68+(green)/iNOS+(red)], M2 [CD68+(green)/Arg-1+(red)] and Treg [CD4+(green)/CD25+(red)/Foxp3+ (far red)]. Scale bar=50µm. **(B–E)** Quantitative analysis of M1, M2, M1/M2 ratio and Treg. **(F–I)** qPCR showed mRNA level of inflammatory cytokines in the heart tissue. Data are presented as mean ± SD. *p < 0.05, **p < 0.01, ***p < 0.001 between groups (One-way ANOVA followed by either the Bonferroni posttest or Two-stage step-up method of Benjamini, Krieger and Yekutieli posttest). iNOS, inducible nitric oxide synthase; Arg-1, Arginase-1. ns, no significance.

The number of infiltrating M1 (CD68+/iNOS+) macrophages was higher in RVH compared to Lean pigs, was normalized in RVH+EVs pigs, but elevated vs. Lean in RVH+IL-10-KD-EVs. In contrast, the number of M2 (CD68+/Arg-1+) macrophages was markedly decreased in RVH and RVH+IL-10-KD-EVs compared to the Lean and RVH+WT-EVs groups. Consequently, the M1-to-M2 ratio, which was higher in RVH compared with Lean, decreased only in RVH+WT-EVs, but not RVH+IL-10-KD-EVs (**Figures 3A–D**). RVH showed a fall in the number of Tregs (CD4+/CD25+/Foxp3+) vs. Lean, whereas their numbers were higher than Lean in RVH+WT-EVs hearts and were lower than RVH+WT-EVs in RVH+IL-10-KD EVs (**Figures 3A, E**).

Expression of the inflammatory cytokine IL-1β mRNA was elevated in RVH and significantly declined in RVH+WT-EVs pigs, while IL-10-KD blunted this anti-inflammatory effect of EVs. IL-6 mRNA expression was not elevated in RVH yet decreased in the WT-EVs-treated group compared to Lean. MCP-1 and TNF-α were upregulated only in RVH+IL-10-KD-EVs (**Figures 3F–I**).

### IL-10-KD-EVs showed impaired capability to attenuate myocardial fibrosis and promote angiogenesis

**Figure 4:**
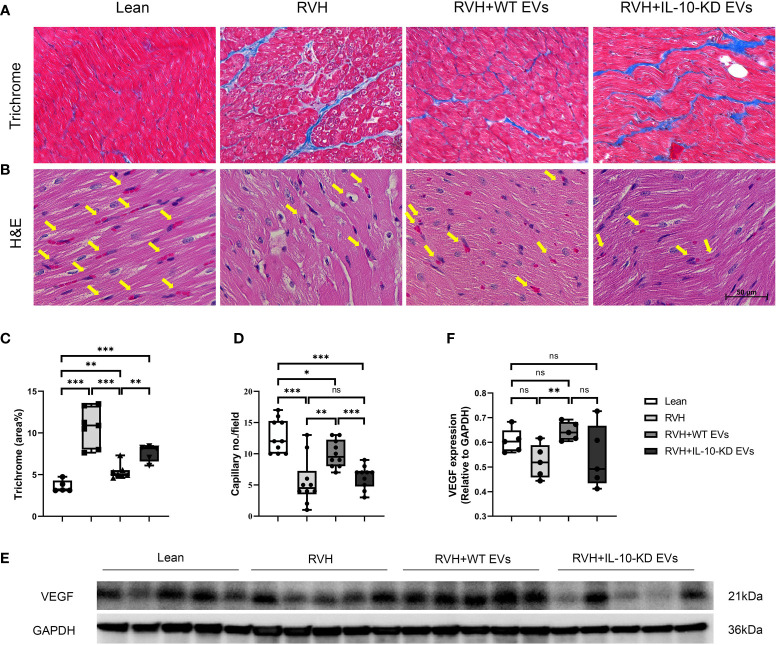
IL-10-KD EVs reversed the effects of WT-EVs on alleviating myocardial fibrosis and increasing capillary density. **(A, C)** Representative Masson’s trichrome staining (40×) and quantification of myocardial fibrosis; **(B, D)** Representative heart sections stained with H&E (40×), showing capillaries (yellow arrows) and quantification of capillary density (numbers/field). **(E, F)** Myocardial protein expression of VEGF and its quantification. Scale bar = 50µm. Data are presented as mean ± SD. *p < 0.05, **p < 0.01, ***p < 0.001 between groups (One-way ANOVA followed by either the Bonferroni posttest or Two-stage step-up method of Benjamini, Krieger and Yekutieli posttest). H&E, Hematoxylin-eosin; VEGF, vascular endothelial growth factor. ns, no significance.

Trichrome staining showed evident myocardial fibrosis in RVH pigs that was significantly blunted by both types of EVs, although IL-10-KD-EVs were less effective than WT-EVs (**Figures 4A, C**).

H&E staining illustrated fewer myocardial capillaries in RVH compared to Lean hearts, which was significantly improved in WT-EVs-treated but not in IL-10-KD-EVs-treated pigs (**Figures 4B, D**).

Myocardial protein expression of VEGF in RVH tended to decrease compared to Lean pigs but did not reach statistical significance (*p*=0.059). Moreover, VEGF expression in RVH+WT-EVs, but not in IL-10-KD EVs, was upregulated compared to RVH (*p*=0.008) (**Figures 4E, F**).

### IL-10-KD-EVs achieved less inhibition of T-cell proliferation than EVs

**Figure 5:**
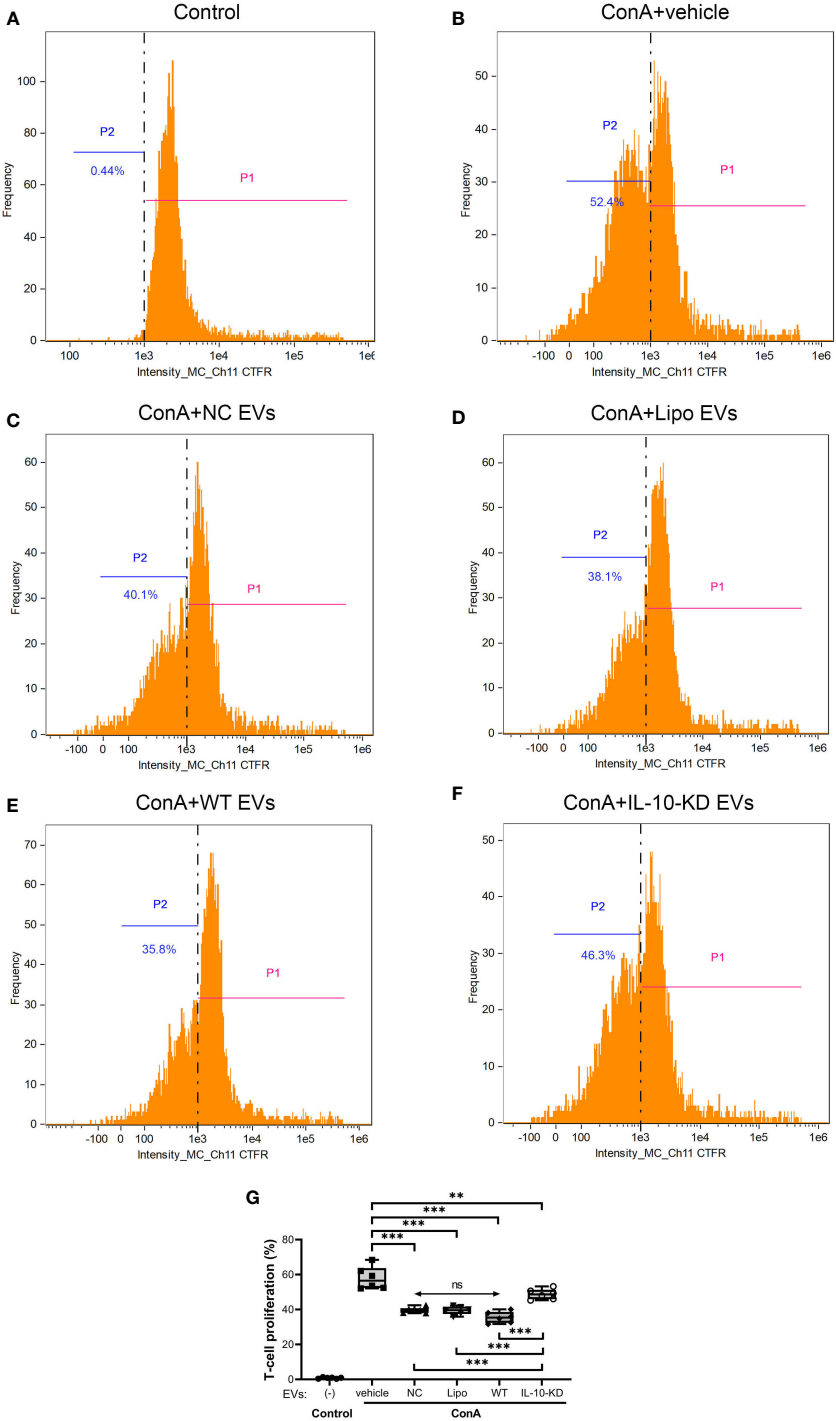
Proliferation ability of naïve CD3+ T-cells cocultured with EVs or IL-10-KD-EVs was assayed using the CTFR method by Flow cytometry (n = 6 biological replicates). **(A)** naïve T-cells without ConA stimulation; **(B)** ConA stimulated the proliferation of T-cells that were then cocultured with **(C)** NC-EVs, **(D)** Lipo-EVs, **(E)** WT-EVs, or **(F)** IL-10-KD-EVs. **(G)** Comparison of proliferation of ConA-stimulated T-cells. Data are mean ± SD. *p < 0.05, **p < 0.01, ***p < 0.001 between groups, double-headed arrow with ‘ns’, no statistical significance among the indicated groups (One-way ANOVA followed by either the Bonferroni posttest or Benjamini, Krieger and Yekutieli posttest). CTFR, CellTrace Far Red; ConA, concanavalin-A; NC, negative control with empty vector; Lipo, lipofectamine, P1, T-cell passage 1, the original T-cell; P2, T-cell passage 2, the proliferated T-cell.

Inhibition of ConA-induced T-cells proliferation was assessed to appraise the immunomodulatory capacity of EVs with or without IL-10-KD. ConA stimulation markedly enhanced the proliferation of naïve T-cells (52.4% vs. 0.44% in control cells, *p*<0.001) (**Figures 5A, B**). NC-EVs, Lipo-EVs, and WT-EVs all attenuated T-cell proliferation to a similar extent (*p*>0.05, (**Figures 5C–E**). IL-10-KD-EVs also inhibited T-cell proliferation (p=0.008 vs. ConA), but less effectively than other EVs (p<0.001, **Figures 5F, G**).

### IL-10-KD-EVs were incapable of stimulating CAEC migration

**Figure 6:**
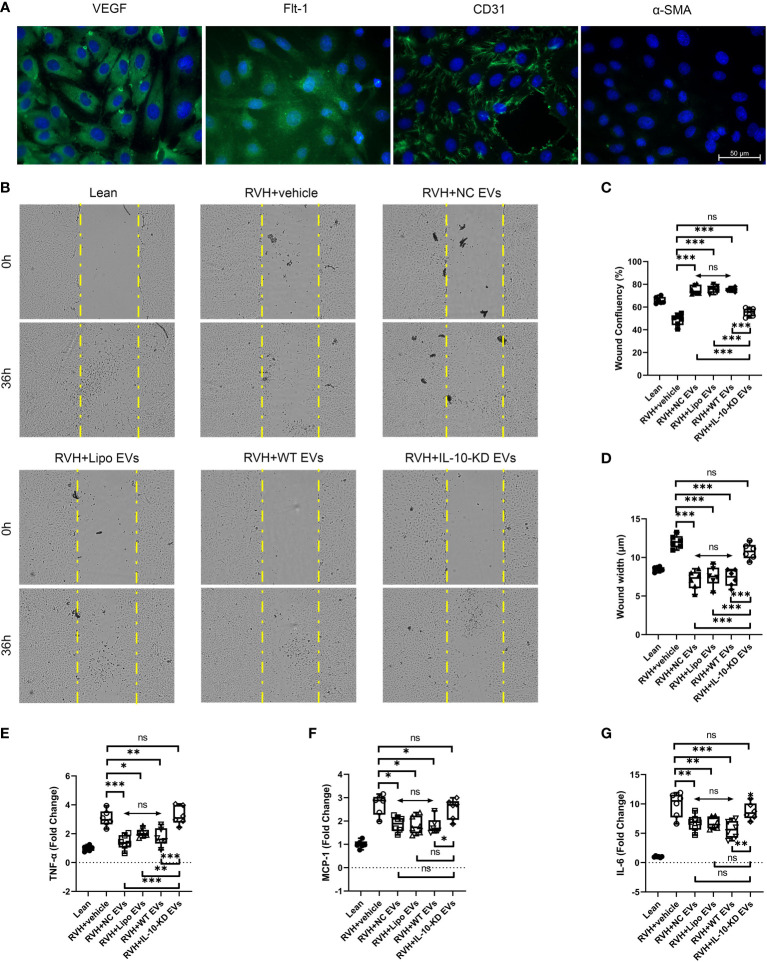
IL-10-KD-EVs failed to stimulate migration or attenuate inflammatory cytokine expression in CAECs. **(A)** Representative immunofluorescence images (40×) for surface markers of CAEC. **(B)** Cell migration ability examined using the wound healing assay under microscopy (10×). **(C, D)** Wound width and confluency quantified at 0h and 36h. **(E–G)** qPCR showed inflammatory cytokines in CAECs cocultured with vehicle, NC-EVs, Lipo-EVs, WT-EVs or IL-10-KD-EVs. Scale Bar = 50µm. *p < 0.05, **p < 0.01, ***p < 0.001 between groups, double-headed arrow with ‘ns’ meant no statistical significance among the included groups (One-way ANOVA followed by either the Bonferroni posttest or Benjamini, Krieger and Yekutieli posttest). CAECs, coronary artery endothelial cells.

To further study the role of IL-10 in regulating the effect of EVs on endothelial cells, primary CAECs were isolated from Lean and RVH coronary arteries and identified as positive for VEGF, Flt-1, and CD31, and negative for α-SMA (**Figure 6A**).

RVH-CAECs showed lower migratory capacity and confluency than Lean-CAECs in the wound-healing assay (**Figures 6B–D**). WT-EVs treatment significantly narrowed the wound width at 36h (7.6µm vs. 12.4µm in RVH, *p*<0.001, **Figure 6C**) and achieved higher CAEC confluency (75.8% vs. 50.7% in RVH, *p*=0.007, **Figure 6D**), whereas IL-10-KD-EVs did not (*p*=0.132 for confluency and *p*=0.181 for width vs. RVH). NC-EVs and Lipo-EVs showed effects similar to WT-EVs.

### IL-10-KD-EVs failed to attenuate inflammatory cytokines released by CAECs

Inflammatory cytokine mRNA levels were also measured in Lean- and RVH-CAECs co-incubated with vehicle, NC-EVs, Lipo-EVs, WT-EVs, and IL-10-KD-EVs. qPCR showed that regardless of co-incubation, TNF-α, MCP-1, and IL-6 were significantly upregulated in all RVH compared to Lean CAECs (**Figures 6E–G**). WT-EVs, NC-EVs, and Lipo-EVs all decreased inflammatory cytokines expression (*p*<0.001), while IL-10-KD blunted this benefit of EVs.

## Discussion

The present study demonstrates that IL-10 content modulates the ability of MSC-derived EVs to improve impaired cardiac diastolic function and tissue injury in experimental RVH. In contrast to WT-EVs, IL-10-KD-EVs incompletely alleviated myocardial fibrosis, inflammation, and capillary loss. Furthermore, IL-10-KD hampered the ability of adipose MSC-derived WT-EVs to restore coronary endothelial cell function or decrease inflammation in pigs with RVH secondary to ischemic and metabolic kidney diseases. These observations highlight IL-10 as an important modulator of the reparative impact of EVs on the cardiac complications of kidney disease.

Early activation of inflammation reflects a reparative response to primary injurious processes. Adaptative repair results in reversible injury and tissue regeneration, whereas maladaptive repair activates local and system inflammatory signaling cascades and causes irreversible damage (26, 27). In pigs with RVH and MetS, we observed significantly increased body weight and GFR secondary to the high-cholesterol/high-carbohydrate diet (14). Stenotic-kidney GFR was lower than in the intact contralateral kidney in RVH, indicating that kidney disease was successfully established. However, because of the compensatory effects of the contralateral kidney, systemic SCr levels were maintained. GFR normalized per unit kidney volume was unchanged among most groups, except being lower in the stenotic kidneys of RVH+IL-10-KD EVs than in RVH, which might due to data variability. These data suggest that the differences in GFR are largely driven by kidney size, which serves to buffer and sustain renal blood flow and GFR per unit kidney volume.

Cardiac injury is a common complication of metabolic and ischemic kidney diseases (28, 29), such as renovascular disease. In our study, the RVH model showed cardiac compensation with pathological changes, including infiltration of inflammatory cells and myocardial fibrosis, with maintained LV systolic but impaired diastolic function, possibly linked to myocardial fibrosis. We have recently shown that treatment with MSC-derived EVs attenuated cardiac dysfunction in this model (13), suggesting that a targeted intervention could delay CVD complications in subjects with metabolic kidney diseases.

MSCs are reparative cells possessing self-renewal, immunoregulatory, and angiogenic capabilities. Previous studies have reported the cardioprotective and renoprotective function of MSCs (30, 31), yet their application is limited by their impurity, consistency, scalability, and potential risks (32). Hence, MSC-derived WT-EVs are considered a safer substitute than their parental cells and carry a complex cargo to exert cell-free effects in recipient cells (33–35).

EVs are bequeathed with the anti-inflammatory and immunomodulatory properties of their parental MSCs. Our study shows that WT-EVs promoted macrophage polarization from an inflammatory M1 to a reparative M2 phenotype, increased the infiltration of reparative Tregs *in vivo*, and decreased the expression of inflammatory cytokines in myocardial tissue, exhibiting their immunoregulatory ability. Similarly, WT-EVs inhibited T-cells proliferation and decreased gene expression of the inflammatory cytokine TNF-α in primary RVH-CAECs *in vitro*. Conversely, IL-10-KD-EVs failed to decrease inflammation either *in vivo* or *in vitro*. IL-10 is a potent anti-inflammatory cytokine with a crucial role in the inflammatory response and injury repair (36–38). Our observations implying that IL-10 is a central mediator of the anti-inflammatory properties of EVs in the myocardium are consistent with previous studies suggesting that IL-10-overexpressing MSCs increased autophagy and protected rats against traumatic brain injury-induced neuronal damage (39). Additionally, MSC-derived EVs alleviated hepatic injury *via* IL-10-mediated M2 Kupffer cell polarization (40). Congruently, M2 reparative macrophages release high levels of anti-inflammatory IL-10 (41).

We also found that both WT and IL-10-KD EVs inhibited T-cell proliferation, yet IL-10-KD EVs were less effective. This property of EVs is inconsistently observed (42). The use of a much higher number of EVs compared to T-cells may account for their notable effectiveness. Trapani et al. reported that bone marrow-MSC-EVs were effective in suppressing B-cell and/or natural killer cell proliferation, but not in inhibiting T-cell proliferation (43), whereas Blazquez et al. showed that adipose tissue MSC-derived EVs were capable of inhibiting T-cell stimulation (44). Additionally, human platelet-derived EVs, isolated by tangential-flow filtration, inhibited T-cell proliferation more potently than EVs isolated by size-exclusion chromatography (21). Taken together, EVs derived from different tissue sources under diverse medium conditions and isolation methods may show variable compositions, resulting in a range of T-cell responses. Nonetheless, our WT-EVs and IL-10-KD-EVs were isolated using the same methodology and should therefore have comparable composition, except for IL-10. Similarly, given that the renal artery stenosis persisted, neither WT-EVs nor IL-10-KD-EVs decreased PRA and MAP, yet WT-EVs improved cardiac histology and inflammatory infiltration.

Besides immunoregulation, WT-EVs are also equipped with the ability to promote angiogenesis, conceivably thanks to their enrichment in proteins that contribute to vascular development (45). WT-EVs improved capillary density and upregulated VEGF expression in cardiac tissue *in vivo*, and promoted the migration of endothelial cells *in vitro*, whereas IL-10-KD impeded this activity. Possibly, the pro-angiogenic capability of IL-10 might be mediated by directly binding and activating its receptor on endothelial cells (46) or by indirectly polarizing macrophages to a reparative phenotype (47). MSCs-secreted IL-10 was implicated in enhancing angiogenesis in transplanted fat tissue (48), and IL-10 promoted ischemia-induced angiogenesis in the retina by altering macrophage angiogenic function (49). Additionally, the pro-angiogenic factor HIF-1α increases IL-10 production by B-cells under hypoxia conditions (50), and IL-10 upregulates the pro-angiogenic angiotensin-converting enzyme-2 in human umbilical vein endothelial cells (51). In our study, IL-10-containing EVs restored VEGF expression and capillary density *in vivo* whereas IL-10-KD impeded the ability of EVs to augment migration and proliferation of endothelial cells. However, we cannot rule out the possibility that the pro-angiogenic property of WT-EVs stems from IL-10-induced macrophage polarization to create an anti-inflammatory and pro-angiogenic microenvironment.

Interestingly, we observed that CAECs obtained from pigs with RVH showed impaired basal confluence and migration capacity as well as increased inflammation consistent with endothelial dysfunction, which improved upon IL-10-containing EV treatment. Additional studies are needed to determine whether EVs restore endothelial function *in vivo*.

Our pre-clinical large animal study revealed that IL-10 partly mediates the effects of EVs on improving cardiac function and decreasing tissue injury in RVH. However, our study also faced some limitations. We did not include NC-EVs and Lipo-EVs *in-vivo*, yet our *in-vitro* studies argue against their direct effects on EVs compared to WT-EVs. Our observation period was relatively short, which might influence the natural history of cardiac dysfunction progression, and treatment might also be more effective during the cardiac compensation phase. We used only female pigs in our study, but these juvenile pigs were pre-menstrual and thus typically not affected by cyclical changes in sex hormone levels. In our previous study using a similar model, SCr significantly increased in RVH compared with Lean pigs, probably due to biological heterogeneity as well as larger group size than in the present study, which might have allowed reaching statistical significance. Nevertheless, a higher SCr in RVH+IL-10-KD-EVs vs. WT-EVs was detected in the current study as well. Furthermore, the timepoint and dose of EV treatment were based on previous studies. The precise mechanisms involved in the influence of IL-10 on the immunoregulation and angiogenesis activities of EVs also remain to be determined.

In conclusion, the present study shows that in RVH pigs myocardial tissue injury precedes cardiac dysfunction, including myocardial fibrosis and infiltration of inflammatory cells. Intra-renal delivery of adipose MSC-derived WT-EVs alleviated myocardial inflammation and fibrosis and increased capillary density *in vivo*, and inhibited T-cells proliferation and CAEC inflammation *in vitro*. Notably, these beneficial effects were blunted with IL-10-KD EVs, suggesting that this anti-inflammatory cytokine contributes to the salutary effects of EVs to prevent cardiac dysfunction and promote angiogenesis. Additional studies are needed to determine if overexpression of IL-10 in EVs augments their function further. IL-10-containing EVs might be an important tool in translational medicine for preventing cardiovascular complications of kidney disease.

## Data availability statement

The original contributions presented in the study are included in the article/Supplementary Material. Further inquiries can be directed to the corresponding author.

## Ethics statement

The animal study was reviewed and approved by Mayo Clinic Institutional Animal Care and Use Committee.

## Author contributions

LL and XZ designed the project. XZ, LZ, SH, and WH maintained the animal models. HT, KJ, IS and YJ performed the morphological, biochemical, and molecular experiments. LL, AL, AE, and YJ interpreted data and created the figures and tables. YJ provided the first draft of the manuscript. All authors contributed to the article and approved the submitted version.

## Funding

This study was partly supported by grants from the NIH (DK120292, DK122734, AG062104 and HL158691). We also thank the China Scholarship Council for support.

## Conflict of interest

LL is an advisor to AstraZeneca, CureSpec, Butterfly Biosciences, Beren Therapeutics and Ribocure Pharmaceuticals.

The remaining authors declare that the research was conducted in the absence of any commercial or financial relationships that could be construed as a potential conflict of interest.

## Publisher’s note

All claims expressed in this article are solely those of the authors and do not necessarily represent those of their affiliated organizations, or those of the publisher, the editors and the reviewers. Any product that may be evaluated in this article, or claim that may be made by its manufacturer, is not guaranteed or endorsed by the publisher.
